# The emerging use of zebrafish to model metabolic disease

**DOI:** 10.1242/dmm.011346

**Published:** 2013-09

**Authors:** Asha Seth, Derek L. Stemple, Inês Barroso

**Affiliations:** 1 Wellcome Trust Sanger Institute, Wellcome Trust Genome Campus, Hinxton, Cambridge, CB10 1SA, UK; 2 University of Cambridge Metabolic Research Laboratories and NIHR Cambridge Biomedical Research Centre, Level 4, Institute of Metabolic Science, Box 289, Addenbrooke’s Hospital, Cambridge, CB2 0QQ, UK

## Abstract

The zebrafish research community is celebrating! The zebrafish genome has recently been sequenced, the Zebrafish Mutation Project (launched by the Wellcome Trust Sanger Institute) has published the results of its first large-scale ethylnitrosourea (ENU) mutagenesis screen, and a host of new techniques, such as the genome editing technologies TALEN and CRISPR-Cas, are enabling specific mutations to be created in model organisms and investigated *in vivo*. The zebrafish truly seems to be coming of age. These powerful resources invoke the question of whether zebrafish can be increasingly used to model human disease, particularly common, chronic diseases of metabolism such as obesity and type 2 diabetes. In recent years, there has been considerable success, mainly from genomic approaches, in identifying genetic variants that are associated with these conditions in humans; however, mechanistic insights into the role of implicated disease loci are lacking. In this Review, we highlight some of the advantages and disadvantages of zebrafish to address the organism’s utility as a model system for human metabolic diseases.

## Introduction

Over the past three decades, the prevalence of the common metabolic diseases obesity, type 2 diabetes (T2D), non-alcoholic steatohepatitis (NASH) and atherosclerosis has soared, and these conditions threaten to diminish lifespan for the first time in the modern era (Wild and Byrne, 2005). Genome-wide association studies (GWAS) and, increasingly, whole-exome and whole-genome sequencing have identified a wealth of candidate human disease genes and loci associated with metabolic disease (reviewed in Travers and McCarthy, 2011). The challenge now is to uncover the role of these genes and determine how their dysfunction affects pathophysiology. Zebrafish have been successfully used in other disease areas to examine variant function *in vivo* at reasonably high throughput, for example in a recent study looking at platelet formation, which has implications for haematological disease (Gieger et al., 2011). Can similar advances be made in the metabolism field utilising the benefits of zebrafish? This Review discusses recent progress in using zebrafish to model the interrelated conditions of the metabolic syndrome, which include obesity, diabetes, fatty liver disease and atherosclerosis.

### Zebrafish as a disease model

Zebrafish have a number of features that make them an attractive research tool. A fundamental advantage is that they share a considerable amount of genetic identity with humans, and several zebrafish organ systems are remarkably similar to those in humans. Moreover, the translucent body of zebrafish embryos facilitates non-intrusive visualisation of organs and biological processes *in vivo*. In combination with this, zebrafish are genetically tractable, amenable to large-scale forward genetic approaches and, as of 2013, their fully sequenced genome is available (Howe et al., 2013). Additionally, zebrafish are relatively inexpensive to maintain, produce large numbers of offspring and undergo rapid development. These advantages reduce both the time and cost of carrying out *in vivo* investigations.

Although zebrafish genetics has traditionally been driven by forward genetic mutagenesis screens (Driever et al., 1996; Haffter et al., 1996), an increasing range of reverse genetic techniques is becoming available. Transient knockdown of gene expression is possible through the use of morpholinos (Nasevicius and Ekker, 2000), which allows rapid and effective study of gene function; however, this approach is limited to processes that occur during the first 5 days of development. In contrast to developmental defects, metabolic phenotypes often become apparent during the larval or juvenile stage, after the embryonic stage; thus, morpholinos are not amenable for studies of metabolic disease. For later-onset phenotypes, fish mutagenised with the chemical ethylnitrosourea (ENU) can be used. Gene-specific mutations can be identified by carrying out loci-specific PCR or, more recently, by whole-exome sequencing, as exemplified by the Zebrafish Mutation Project (Kettleborough et al., 2013) (Box 1). A further advance has been the use of transcription activator-like effector nucleases (TALENs) (Sander et al., 2011) and the CRISPR-Cas system (Hwang et al., 2013) to create targeted mutations. Both of these methods introduce locus-specific double-strand breaks in the genome, generating disruptive mutations with relative ease. TALENs consist of a sequence-specific DNA-binding domain fused to a nonspecific DNA cleavage module. The DNA-binding domain can be customised to recognise virtually any sequence using a modular recognition code that is bound by TALE proteins, a group of naturally occurring proteins found in plant bacteria (Boch et al., 2009). The CRISPR-Cas system uses a guide RNA molecule whose sequence is complementary to part of the gene of interest to programme a nuclease to target one or more positions in the genome. Both TALEN and CRISPR-Cas genome editing techniques can be utilised to precisely modify the sequence at a particular locus by the addition of template DNA sequences, thus allowing specific genetic variants to be modelled (Bedell et al., 2012; Chang et al., 2013). Transgenic techniques have also been improved, most notably with the creation of constructs that allow conditional gene activation or inactivation (Ni et al., 2012). Thus, we are entering an era in which specific human mutations can be modelled in a temporally and spatially specific way in the zebrafish.

Box 1. **The Zebrafish Mutation Project**

The Zebrafish Mutation Project (ZMP) was launched in 2011 as an initiative to create a knockout allele in every protein-coding gene in the zebrafish genome. The project involves sequencing of exon-enriched DNA from first-generation offspring (F1) produced from a mating of an ENU-mutagenised male with a wild-type female and then identification of induced mutations using a modified version of the 1000 Genomes Project variant-calling pipeline (Abecasis et al., 2010). Each allele with either a nonsense or essential splice mutation is analysed for morphological differences during the first 5 days of development. As of June 2013, the sequence of 2304 F1 individuals had been analysed and 24,622 nonsense and essential splice alleles in 12,166 genes had been identified. Therefore, the ZMP currently has mutant models for 46% of all protein-coding genes. All the data generated can be accessed on the ZMP website (http://www.sanger.ac.uk/Projects/D_rerio/zmp/), and alleles are archived and can be requested from the Zebrafish International Resource Center (ZIRC).

**Figure:**
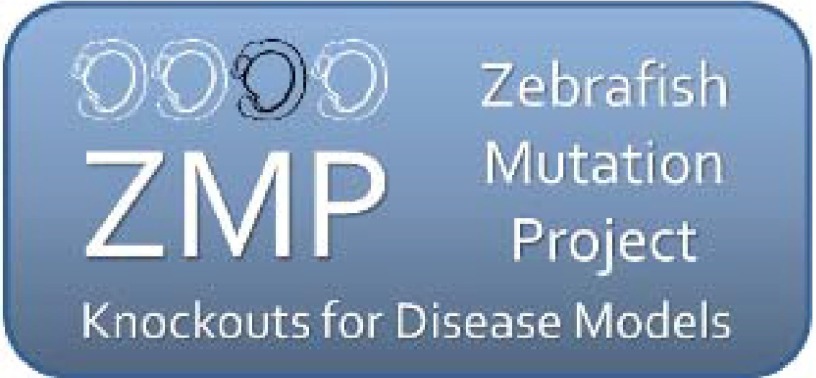

An exciting new development is the establishment of a transcript counting (TC) pipeline to complement the morphological analysis. The TC identifies differentially expressed transcripts between mutant embryos and sibling controls using a 3’ end based RNAseq protocol. This analysis is limited to those mutant alleles that show a morphological or behavioural phenotype.

 

### Modelling metabolic disease using zebrafish

Metabolic control and regulation of whole-body energy homeostasis involve a complex interplay between multiple organs and endocrine signals to carefully balance energy intake, utilisation and storage. *In vitro* studies cannot recreate the complexity that exists *in vivo*, so whole-animal approaches are required to study metabolism as it plays out in a multicellular context. Zebrafish are a good model in which to study metabolism because they possess all the key organs required for metabolic control in humans, from the appetite circuits that are present in the hypothalamus, through to the pancreas and insulin-sensitive tissues [liver, muscle and white adipose tissue (WAT)].

In contrast to mice and humans, zebrafish are poikilothermic (body temperature varies with the ambient temperature), and brown adipose tissue (BAT) depots have not been identified. Therefore, the study of pathways that are activated by non-shivering thermogenesis, for example the β-adrenergic system, will be more limited in zebrafish than in mice. On the other hand, most standard animal facilities house small rodents at an ambient temperature substantially below the temperature associated with thermoneutrality in mice; thus, metabolic analysis is routinely carried out using animals that are chronically challenged by cold stress. This condition induces a range of physiological responses – such as increased food intake, metabolic rate and sympathetic activity – that could mask the metabolic phenotype under analysis (Overton, 2010). The dramatic effect of ambient temperature on metabolic phenotypes in mice is clearly illustrated by the uncoupling protein 1 gene (*Ucp1*) knockout mouse. Ucp1 is a mitochondrial protein that is specifically expressed in BAT and is required for the dissipation of energy and production of heat by non-shivering thermogenesis. Ectopic expression of Ucp1 in skeletal muscle prevents diet-induced obesity and insulin resistance in mice (Li et al., 2000), and it was anticipated that lack of *Ucp1* would lead to an obese phenotype. Surprisingly, original studies of *Ucp1*-ablated mice failed to demonstrate any significant change in body mass (Enerbäck et al., 1997). However, when the mice were maintained in a thermoneutral environment, an obese phenotype was observed (Feldmann et al., 2009). This highlights the potential for thermogenesis-associated bias in studies of metabolic disease, and suggests that the zebrafish could provide an excellent additional model to circumvent the problem. Used in combination with mammalian models, the unique features of the zebrafish provide a powerful tool for dissecting metabolic disease, as illustrated in the rest of this Review and summarised in Table 1.

**Table 1.**
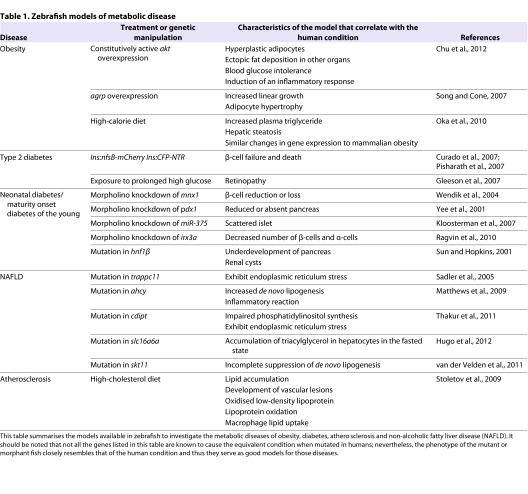
Zebrafish models of metabolic disease

## Using zebrafish to study obesity

The hypothalamic circuitry, which regulates energy balance in vertebrates, is largely conserved between humans and zebrafish. As in humans, in zebrafish, the leptin receptor and proteins of the melanocortin system are expressed in the hypothalamus (Liu et al., 2010; Zhang et al., 2012), and intracerebroventricular administration of the neuroactive peptides NPY (neuropeptide Y), ghrelin and AgRP (Agouti-related peptide) stimulates food intake, whereas administration of CART (cocaine and amphetamine regulated transcript) peptide, melanocortin and CRF (corticotropin-releasing factor) inhibit feeding (Kawauchi, 2006). In humans, mutations in melanocortin receptor 4 (*MC4R*) are the most common genetic cause of monogenic, or single-gene, obesity (Farooqi and O’Rahilly, 2006). The consequence of loss of *mc4r* on body mass in zebrafish is currently not known; however, transgenic overexpression of AgRP, the endogenous inverse agonist for Mc4r, results in obese zebrafish that exhibit increased linear growth and adipocyte hypertrophy, consistent with the hypothesised role of Mc4r (Song and Cone, 2007). The conserved neural circuitry opens the door to the possibility of carrying out *in vivo* imaging of hypothalamic neuronal activation using genetically encoded fluorescent Ca^2+^ indicators targeted to distinct neural populations, in particular in mutant zebrafish displaying altered food intake. This technique has been previously used to study hypocretin-positive neurons (a population of approximately only 20 neurons) in awake, freely behaving zebrafish. The authors observed a bioluminescent signal from the reporter that was associated with periods of increased locomotor activity, in line with the role of this neuronal population in regulating arousal, wakefulness and appetite (Naumann et al., 2010).

**Fig. 1.**
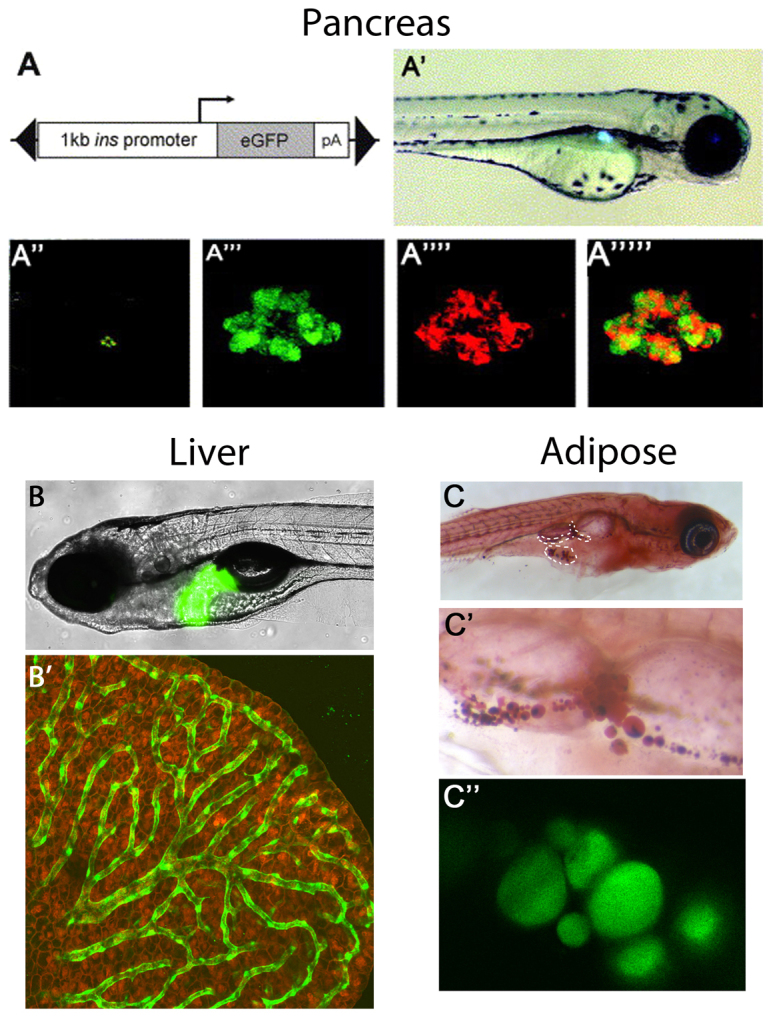
**Visualisation of metabolic tissues in zebrafish.** (A–A″″′) Pancreas. The insulin (*ins*) promoter drives the expression of GFP in β-cells. (A) Schematic of construct used. (A′) Fluorescent image of a 3-day-old *ins:EGFP* embryo showing expression within the islet of the pancreas. (A″) Rendered composite of confocal images of a 10-day-old *ins:EGFP* embryo following immunofluorescence to detect insulin, with overlaid 20× brightfield. (A″′–A″″′) Close-up images of EGFP fluorescence (A″′), immunofluorescence to detect insulin (A″″) and merged image demonstrating colocalization of EGFP with insulin (A″″′). Reproduced with permission (Pisharath et al., 2007). (B,B′) Liver. (B) A transgenic zebrafish that allows researchers to visualise glucose production in the liver. A 2.8-kb fragment of the phosphoenolpyruvate carboxykinase (*pck1*) promoter drives expression of a fluorescent protein (venus). Reproduced with permission (Gut et al., 2012). (B′) Confocal analysis of the liver parenchyma of a 1.5-month-old live *LiPan/Tg[fli:EGFP* transgenic zebrafish. Hepatocytes express the dsRed RFP reporter gene under a liver-specific *lfabp* promoter and the vasculature is labelled in green. Reproduced with permission (Korzh et al., 2008). (C–C″) Adipose. Low (C) and high (C′) power image of juvenile fish stained with Oil Red O, indicating the location of the adipocytes close to the intestine and pancreas. (C″) Confocal imaging of lipid droplets in juvenile fish stained with LipidTox. Images are authors’ own.

In zebrafish, similar to mammals, excess nutrients are stored in the form of large unilocular lipid droplets in white adipocytes, suggesting that the zebrafish could serve as a useful model in which to study the biology of the adipose depot itself. This is in contrast to *Drosophila* and *Caenorhabditis elegans*, where fat is stored in non-specialised cells (within the fat body or the intestine, respectively) that carry out several other functions in addition to lipid storage (Gesta et al., 2007). In zebrafish, the first adipocytes appear in association with the pancreas ∼12 days post-fertilisation (dpf). Pancreatic WAT appearance and quantity correlates with fish length rather than age (Imrie and Sadler, 2010), and adipocytes can by visualised and quantified by staining with the neutral dye Oil Red O or with Nile red, a lipophilic stain that fluoresces in lipid-rich environments (Flynn et al., 2009; Minchin and Rawls, 2011) (Fig. 1C). The advantage of fluorescent staining is that it can be used in live fish, allowing real-time imaging of the formation of adipocytes and their expansion under conditions of nutrient excess. A transgenic reporter line for adipose tissue would provide an invaluable tool for researchers interested in adipose biology and, if used in combination with a model of obesity, would be an excellent resource for high-throughput screening of potential drugs for the treatment of obesity.

Depending on the promoter used, reporter strains might also be useful in gaining a greater understanding of the steps leading to adipocyte cell commitment. Adipocyte differentiation can be viewed as a two-step process: (1) commitment to the adipogenic fate by mesenchymal stem cell progenitors and (2) induction of the adipocyte gene expression programme, which drives terminal differentiation. The pathways regulating the fate choice between adipocyte, osteoblast, myocyte and chondrocyte in step 1 remain unclear, and studies investigating this would benefit from the availability of novel fluorescent reporter lines for carrying out lineage-tracing studies *in vivo*. The adipose vasculature has been proposed to function as a progenitor niche and might provide the signals for adipocyte development (Tang et al., 2008). It would therefore be interesting to study the process of adipocyte commitment in the context of a vascular reporter line such as *fli1:EGFP*. In this strain the friend leukaemia integration 1 transcription factor (*fli1*) promoter drives expression of enhanced green fluorescent protein (EGFP) in all blood vessels throughout embryogenesis, and this line has been previously used in conjunction with time-lapse multiphoton laser scanning microscopy to directly observe angiogenesis (Lawson and Weinstein, 2002).

In contrast to step 1, the primary drivers of adipocyte gene induction have been well characterised, by using human 3T3L1 immortalised cells. The key drivers include peroxisome proliferator-activated receptor-γ (PPARγ), CCAAT/enhancer binding protein α (C/EBPα), C/EBPβ and C/EBPδ (Farmer, 2006). The zebrafish orthologues of these genes are expressed in adipocytes but also in the liver, which is reminiscent of the non-tissue-specific expression observed in mammals (Imrie and Sadler, 2010). Terminal differentiation markers include fatty acid binding protein 4 (FABP4), glucose transporter 4 (GLUT4), leptin and adiponectin (Rosen and MacDougald, 2006). The zebrafish orthologue of *FABP4* is *fabp11a*, and there is evidence that it is expressed in preadipocytes (Flynn et al., 2009) and adult adipose tissue (Imrie and Sadler, 2010); thus, tracing studies using the *fabp11a* promoter would be of great interest. In zebrafish, adiponectin is selectively expressed in adult adipose depots (Imrie and Sadler, 2010); in contrast, the leptin homologue shows low conservation with its mammalian counterparts and does not seem to be adipose-specific: highest levels of this protein are found in the liver (Pfundt et al., 2009). The Glut4 orthologue has not been studied. More recently, the zinc-finger protein Zfp423 was identified as a preadipocyte marker in mice, produced by cells that reside in the adipose vasculature (Gupta et al., 2012). Studies in zebrafish involving live imaging of the proliferation of this progenitor population would be of great interest.

The reasons behind the functional and biological differences between subcutaneous and visceral adipose depots are another unresolved issue in adipose biology. Subcutaneous fat is located beneath the epidermis, whereas visceral fat is located within the peritoneal cavity close to the endodermal organs of the abdomen. It is not clear whether it is the location of visceral fat and its exposure to particular paracrine or endocrine signals that determines its properties or if distinct developmental programmes drive subcutaneous and visceral adipocyte differentiation. It is also not understood why the health consequences of excess subcutaneous and visceral adipose tissue differ, with increased metabolic and cardiovascular risk being associated with the latter but not the former (Gastaldelli et al., 2002; Vega et al., 2006). In humans (and rodents), it is difficult to measure the relative amounts of each of these depots without expensive imaging procedures. *In vivo* imaging of changes in subcutaneous and visceral WAT depots in the zebrafish during development and under different metabolic challenges might help to provide insight into the intrinsic differences between these two adipose depots.

## Using zebrafish to study diabetes

Zebrafish have been used extensively to study pancreas development. The pancreas in zebrafish is comprised of exocrine and endocrine compartments connected by a ductal system to the digestive tract, as in mammals. Zebrafish pancreatic islets consist of a central core of insulin-producing β-cells surrounded by glucagon-producing α-cells, δ-cells (which produce somatostatin) and ε-cells (which produce ghrelin). The primary islet can be identified as early as 24 hours post-fertilisation (hpf) (Argenton et al., 1999), and the secondary islets begin to form at 5 dpf (Hesselson et al., 2009). The latter constitute the major part of the adult pancreas. Several genes have been shown to influence β-cell development when perturbed in zebrafish (Kinkel and Prince, 2009) (see Table 1) and, in some cases, the resulting phenotype closely mimics those associated with established human diseases. For example, a mutation in Hnf1β results in underdevelopment of the pancreas and development of kidney cysts (Sun and Hopkins, 2001), similar to the human condition maturity-onset diabetes of the young (MODY) type 5 (Fajans et al., 2001).

Zebrafish strains in which the pancreatic β-cells can be visualised with the use of a fluorescent reporter (Fig. 1A) and conditionally ablated by the addition of a prodrug [metronidazole (Mtz)] are available. In these transgenic lines, nitroreductase (NTR) and the fluorescent reporter are placed under the control of the insulin promoter. NTR is a bacterial enzyme that catalyses the reduction of the innocuous prodrug Mtz into a cytotoxic product that induces cell death. Following exposure to the prodrug, NTR instigates ablation of the β-cell. After removal of the drug, reconstitution of β-cell mass can be achieved in just 35 hours (Curado et al., 2007; Pisharath et al., 2007), enabling studies of β-cell regenerative capacity (Table 1). Recently, Andersson and colleagues utilised this model to carry out a high-throughput screen for drugs that enhance β-cell regeneration (Andersson et al., 2012). Their hits were subsequently validated in mice, further confirming the similarity between zebrafish and mammalian biology.

Quantitative analysis of glucose homeostasis can be carried out in mature zebrafish via whole-blood analysis (Moss et al., 2009). However, the small size of embryos and juvenile fish limits the suitability of this method, particularly in studies of a high-throughput nature. An alternative method involves measurement of absolute glucose levels from embryo extracts (Jurczyk et al., 2011) using a dual enzyme fluorescent assay that detects free glucose, i.e. glucose that has not been phosphorylated intracellularly by hexokinases. Andersson and colleagues demonstrated an ∼threefold increase in free glucose levels following targeted β-cell ablation, suggesting that this method could be useful for quantifying islet functionality (Andersson et al., 2012).

Recently, another transgenic zebrafish line was developed to facilitate the identification of new modulators of glucose homeostasis. Gut and colleagues generated a transgenic bioluminescent and fluorescent reporter line in which reporter expression is under the control of the phosphoenolpyruvate carboxykinase (*pck1*) promoter [*Tg(pck1:Luc2)*] (Gut et al., 2012) (Fig. 1B). Pck1 is a hepatic enzyme, induced in the fasting state, that catalyses the rate-limiting step of gluconeogenesis, the process in which glucose is synthesised. Gut et al. confirmed that the *Tg(pck1:Luc2)* line faithfully recapitulates the endogenous regulation of Pepck expression in mammals by demonstrating a robust increase in bioluminescence in *Tg(pck1:Luc2)* zebrafish in the fasting state and the appropriate response to pharmacological modulation. Gut et al. then went on to test 2400 bioactive compounds for their ability to modulate *pck1* promoter activity. They also measured whole-larva glucose levels for 60 of these compounds. They identified two novel compounds capable of lowering blood glucose, but these compounds also paradoxically activated *pck1* expression. Gut et al. reasoned that the increased *pck1* expression observed was a compensatory gluconeogenic response to the low glucose levels induced by these molecules and suggested that the two molecules induced a fasting-like metabolic state. Investigation of one of these compounds found that it is able to protect against diet-induced hepatic steatosis and glucose intolerance in mice. Thus, by using this transgenic zebrafish line the authors were able to identify a novel drug that might be useful in the treatment of metabolic dysregulation. Future studies to uncover molecules that can lower Pck1 expression or that act synergistically with known modulators of *pck1* will be of value.

The zebrafish has also been used to study the process of β-cell neogenesis. In a recent study by Maddison and Chen, the authors found that continuous administration of both a glucose-rich diet and a lipid-rich diet for 8 hours can induce β-cell neogenesis in 6-dpf larvae (Maddison and Chen, 2012). The glucose-induced effects were mediated by mammalian target of rapamycin (mTOR), whereas lipid-induced neogenesis required insulin–IGF-1 signalling. These studies suggest that the mTOR and insulin pathways are acting as nutrient sensors that, when activated, can promote β-cell neogenesis. It would be interesting to examine the requirement and function of metabolic cues in other settings of β-cell generation. The overnutrition regime used in this study provides a useful system for inducing T2D in the non-mammalian model. However, to truly recapitulate the human condition it will be necessary to demonstrate some degree of β-cell failure and/or death. Maddison and Chen failed to detect β-cell apoptosis after 4 days of overnutrition; however, it is possible that an extended period of overnutrition is required to see this effect.

Zebrafish have also been used in a chemical screen to identify small molecules that are capable of inducing β-cell neogenesis (Rovira et al., 2011). In this study, Rovira and colleagues looked for small molecules that could induce precocious secondary islet differentiation in the zebrafish pancreas. They identified six hits, three of which had not previously been linked to β-cell differentiation. Collectively, these studies highlight the key advantages of the zebrafish model for researchers within the diabetes field: the ability to modulate β-cell number, visualise β-cell genesis and replication, and assess β-cell function using the glucose assay. Used in combination with high-throughput screening of small-molecule libraries, the zebrafish can be a powerful new tool for diabetes drug discovery.

## Using zebrafish to study fatty liver disease

Excessive accumulation of lipids in the liver can lead to a spectrum of disorders that encompass inflammation, fibrosis and cancer. These interrelated conditions are collectively known as non-alcoholic fatty liver disease (NAFLD). Currently, apart from its association with obesity and insulin resistance, relatively little is known about what triggers and what drives the progression of NAFLD. In the zebrafish, the formation of the liver primordium and the differentiation of hepatocytes and cholangiocytes can be observed by 48 hpf (Chu and Sadler, 2009). The depletion of the yolk and the initiation of independent feeding seems to trigger the activation of several metabolic pathways in the liver and, between days 4 and 6, key genes involved in liver metabolism such as *uncoupling protein 2*, *pck1* and *carnitine palmitoyltransferase 1A* are readily detectable by quantitative reverse transcriptase PCR (qRT-PCR). The relative expression levels of these genes is similar to those seen in the adult mouse liver, suggesting that by day 6 the zebrafish liver is functionally mature, at least in terms of metabolism (Gut et al., 2012). A fatty liver can be detected in zebrafish by whole-mount staining with Oil Red O (Sadler et al., 2005). Lipid uptake from the intestine can also be visualised in real time using fluorescently labelled fatty acid analogues (Farber et al., 2001). Screening of ENU mutants by using fluorescent analogues revealed a mutant with impaired digestive lipid processing. Further work identified that the mutated gene encodes a newly identified protein that is involved in endoplastic reticulum (ER)-Golgi trafficking (Ho et al., 2006). This study demonstrates how a forward genetics approach can identify proteins that have not previously been associated with the process under study.

A number of hepatic steatosis zebrafish mutants have been identified (reviewed in Schlegel, 2012) (Table 1). A common thread that can link genes that have diverse biochemical functions is the role of ER stress in generating hepatic steatosis (Cinaroglu et al., 2011; Sadler et al., 2005; Thakur et al., 2011). One particularly interesting model is the *ducttrip* line, which harbours mutations in *s-adenosylhomocysteine hydrolase* (*ahcy*), a metabolic enzyme that is required to produce methyl donors for use in numerous biological processes. Mutant fish lacking *ahcy* develop hepatic steatosis and liver degeneration (Matthews et al., 2009). Humans with a rare genetic disorder caused by AHCY deficiency display liver dysfunction and a spectrum of impairments in brain function ranging from delayed psychomotor development to hypotonia, feeding problems and respiratory failure leading to death (Baric et al., 2004; Grubbs et al., 2010). Therefore, the zebrafish mutant could be a useful model to study the pathophysiology of this very serious condition. In line with this, morpholino knockdown of tumour necrosis factor α in *ahcy* zebrafish mutants reverted hepatic steatosis and liver degeneration, highlighting new potential therapeutic options for individuals with AHCY deficiency (Matthews et al., 2009).

## Using zebrafish to study atherosclerosis

The formation of atheromatous plaques within arteries leads to the development of atherosclerosis. Atherosclerosis is closely linked with high plasma lipid levels and ensuing vascular inflammation. The induction of extreme hyperlipidaemia in rodents has enabled modelling of the disease and its progression. In zebrafish, a high-cholesterol diet (HCD) can induce the same pathophysiology (see Table 1), with the added benefit that the temporal course of pathogenic events can be monitored in the same animal *in vivo* via the use of fluorescent reporters and live imaging. For example, feeding *fli1:EGFP* transgenic fish with an HCD laced with a fluorescent lipid tracer has allowed researchers to visualise lipid accumulation in the vascular wall (Stoletov et al., 2009). Additionally, use of the transgenic line *lyz:Ds Red*, which labels macrophages and granulocytes, allows the recruitment of myeloid cells in the vasculature to be monitored. As confirmation of the usefulness of this model for testing novel anti-dyslipidaemia drugs, Baek and colleagues verified that, as in humans, ezetimibe, a synthetic compound used to treat hypercholesterolaemia by inhibiting the absorption of cholesterol from the intestine, effectively reduces cholesterol levels in zebrafish that are fed an HCD (Baek et al., 2012).

The *lyz:Ds Red* line could also be used to study macrophage infiltration into adipose depots in zebrafish subjected to an overfeeding regime. This would allow *in vivo* monitoring of the early pathogenic processes of obesity, in particular the inflammatory response and its consequence on adipose tissue morphology and function. Many studies have implicated a role for inflammation in obesity (reviewed in Bastard et al., 2006); however, the direction of causality remains unclear. This study would be very powerful if at the same time an assessment of insulin resistance in metabolic tissues could be carried out, with lipid accumulation in the liver perhaps being used as a surrogate measure. These studies would then help us to elucidate whether inflammatory activation is a cause or consequence of insulin resistance and metabolic disease, and enable identification of the point at which healthy adipose tissue remodelling in the form of adipocyte hypertrophy and hyperplasia becomes maladaptive and pathological.

More generally, the zebrafish is a good model for studying the relationship between oxidative processes, inflammation and chronic disease. Several studies postulate that, during obesity, the increase in adipose mass and adipocyte size leads to an inadequate supply of oxygen and nutrients, leading to a hypoxic and pro-inflammatory environment that promotes insulin resistance. In support of this theory, overexpression of vascular endothelial growth factor in adipose tissue in mice increases blood vessel number and size, and protects against diet-induced obesity and insulin resistance (Elias et al., 2012). Zebrafish express homologues of the crucial components of the mammalian oxygen-sensing signalling system, including hypoxia-inducible factor (HIF), the von Hippel-Lindau protein (pVHL) and several isoforms of prolyl hydroxylase (PHD). A transgenic reporter line has been generated that changes from yellow to red in the presence of H_2_O_2_, enabling *in vivo* study of reactive oxygen species (ROS). This line has been used to study the spatiotemporal gradients of H_2_O_2_ in wound response (Niethammer et al., 2009). A hypoxia-responsive transgenic reporter line, *Tg(phd3:EGFP)*, has also been generated. Experiments with this line revealed that fish with a mutation in the tumour suppressor gene *pVHL* display a systemic hypoxia response (Santhakumar et al., 2012). The use of the H_2_O_2_ or the hypoxia reporter in the context of fish models for both obesity and T2D (Table 1) would provide invaluable insight into the contribution of either oxygen deprivation or damaging oxidative by-products to disease progression throughout the course of the pathological processes underlying chronic disease.

## Future directions and remaining challenges

The findings discussed above illustrate that several metabolic diseases have been successfully modelled in the zebrafish. For diseases affecting β-cell function in particular, we have seen how these models can be utilised with great success to visualise dynamic cell processes and in high-throughput small-molecule screens. The emerging zebrafish models within the disease areas of obesity, NAFLD and atherosclerosis are primed to maximise the advantages of this model system and mimic the advances made within the diabetes field. The development of additional genetic tools such as fluorescent reporter lines for adipose tissue and the different hypothalamic cell types along with improved tools for interrogating insulin signalling would help the zebrafish model reach its full potential within the metabolism field.

Another active area of research within the zebrafish community is the characterisation of non-coding regulatory elements of human disease genes. In enhancer screens, putative enhancer regions drive the expression of a fluorescently labelled molecule to enable the dissection of regulatory regions of the genome. The imaging process can be automated to allow for high-throughput analysis (Gehrig et al., 2009). Only 12% of the single-nucleotide polymorphisms (SNPs) identified from GWAS lie inside transcribed regions. The remaining SNPs lie within non-coding introns or are intergenic, and it is likely that the underlying mechanisms linking these hits to the phenotype are regulatory (Manolio, 2010). Enhancer characterisation studies in zebrafish used in combination with regulatory information identified in the ENCODE project (Dunham et al., 2012) will provide invaluable information to help understand how these loci might contribute to human disease. The impact of non-coding mutations on human disease has been clearly illustrated by the identification of five recessive mutations in the regulatory region of the *INS* gene that result in permanent neonatal diabetes (PNDM) (Garin et al., 2010). The zebrafish could also be useful in determining the role of microRNAs in the regulation of energy and glucose homeostasis. For example, Kloosterman and colleagues demonstrated that miRNA-375 is necessary for normal islet clustering and formation in the zebrafish (Kloosterman et al., 2007) (Table 1). Subsequent studies showed that loss of miR-375 in mice leads to a reduction in β-cell number (Poy et al., 2009).

The small size of the zebrafish does hamper some types of metabolic investigation; for example, intraperitoneal and intracerebroventricular injections, although possible in adult zebrafish (Kinkel et al., 2010; Yokobori et al., 2011), are technically more challenging than the same procedures in larger species such as mice. In addition, blood sampling to measure metabolite or hormone levels is generally a terminal procedure so repeated sampling is not possible. This limitation increases the number of fish required for metabolic studies and prevents analysis of disease progression by biochemical means. Also, it is not clear how many of the radioimmunoassays and enzyme-linked immunosorbent assays (ELISAs) currently available for use with rodent samples will also cross-react with zebrafish serum; a centralised and open-access database outlining zebrafish-compatible assays would be an invaluable resource to the zebrafish community. Furthermore, the current techniques to measure food intake (Volkoff and Peter, 2006) and energy expenditure (Makky et al., 2008) in the zebrafish are not as sophisticated as those in rodents, meaning that subtle metabolic phenotypes might not be detectable. Nevertheless, acid production has been used as a surrogate measure of metabolic rate and was successfully used to demonstrate that *lkb1* mutant zebrafish exhibit an increased metabolic rate (van der Velden et al., 2011) (Table 1). These limitations should be considered when planning metabolic studies in zebrafish.

Another point to consider is the high genetic diversity observed between individual zebrafish genomes, even within fish of the same strain (Guryev et al., 2006). To overcome this, Shinya and Sakai set out to generate the first official inbred zebrafish strain (in-crossed at least 20 times). As part of this endeavour, they recently established the India Strain (IM), which has endured 16 generations and has maintained a healthy condition (Shinya and Sakai, 2011). To reduce the variability induced by multiple polymorphic modifiers, it might be advantageous to carry out studies that involve metabolic phenotyping on this inbred line where possible in the future.

Collectively, there are many exciting possibilities emerging that use zebrafish to model human metabolic disease. It is envisaged that the unique tools provided by the zebrafish system will advance our understanding of metabolic disease onset and progression, and identify currently unknown targets for disease treatment.
